# Blood volume expansion, normovolemia, and clinical outcomes in chronic human heart failure: more is better

**DOI:** 10.1152/ajpheart.00336.2021

**Published:** 2021-10-22

**Authors:** Wayne L. Miller, John E. Strobeck, Diane E. Grill, Brian P. Mullan

**Affiliations:** ^1^Department of Cardiovascular Medicine, Heart-Lung Center, Hawthorne, New Jersey; ^2^Heart-Lung Center, Hawthorne, New Jersey; ^3^Department of Biomedical Statistics and Informatics, Mayo Clinic, Rochester, Minnesota; ^4^Division of Diagnostic Radiology, Mayo Clinic, Rochester, Minnesota

**Keywords:** blood volume expansion, chronic heart failure, outcomes, plasma volume, RBC mass

## Abstract

Expansion in blood volume (BV) is a well-recognized response to arterial underfilling secondary to impaired cardiac output in heart failure (HF). However, the effectiveness of this response in terms of outcomes remains inadequately understood. Prospective analysis was undertaken in 110 patients with HF hospitalized and treated for fluid overload. BVs were measured in a compensated state at the hospital discharge using the indicator-dilution methodology. Data were analyzed for composite 1-year HF-related mortality/first rehospitalization. Despite uniform standard of care, marked heterogeneity in BVs was identified across the cohort. The cohort was stratified by BV expansion greater than or equal to +25% above normal (51% of cohort), mild-moderate expansion (22%), and normal BV (27%). Kaplan–Meier (K-M) survival estimates and regression analyses revealed BV expansion (greater than or equal to +25%) to be associated with better event-free survival relative to normal BV (*P* = 0.038). Increased red blood cell mass (RBCm; RBC polycythemia) was identified in 43% of the overall cohort and 70% in BV expansion greater than or equal to +25%. K-M analysis demonstrated polycythemia to be associated with better outcomes compared with normal RBCm (*P* < 0.002). Persistent BV expansion to include RBC polycythemia is common and, importantly, associated with better clinical outcomes compared with normal total BV or normal RBCm in patients with chronic HF. However, compensatory BV expansion is not a uniform physiological response to the insult of HF with marked variability in BV profiles despite uniform standard of care diuretic therapy. Therefore, recognizing the variability in volume regulation pathophysiology has implications not only for impact on clinical outcomes and risk stratification but also potential for informing individualized volume management strategies.

**NEW & NOTEWORTHY** The novel findings of this study demonstrate that intravascular volume profiles among the patients with chronic heart failure (HF) vary substantially even with similar clinical compensation. Importantly, a profile of blood volume (BV) expansion (compared with a normal BV) is associated with lower HF mortality/morbidity. Furthermore, RBC polycythemia is common and independently associated with improved outcomes. These observations support BV expansion with RBC polycythemia as a compensatory mechanism in chronic HF.

## INTRODUCTION

The importance of an effective blood volume (BV) to maintain integrity of the circulatory system in health and disease is well recognized. The circulation will fail if intravascular volume is insufficient to fill the capacity of the vascular system. With the onset of heart failure (HF) and associated impaired cardiac output, arterial underfilling develops and, as a result, compensatory mechanisms are activated to maintain intravascular volume and critical perfusion pressure despite hemodynamic perturbations. Sympathetic-mediated arterial and venous constriction provide early responses to maintain organ perfusion; however, longer-term mechanisms also come into play that include an expansion in intravascular volume. The concept of BV expansion in patients with chronic HF is not new and has a background in earlier human physiology investigations demonstrating that both intravascular and interstitial compartment fluid volumes increase in HF and that fluid volume distributions can be highly variable (1–5). We and others have shown, by quantitative volume analyses, not only persistence in intravascular volume expansion despite aggressive diuretic therapy but also, and importantly, significant variability in intravascular volume profiles (normal BV to marked expansion) despite similar clinical presentations and treatment (6–10). Therefore, whether BV expansion provides the optimal volume to help maintain circulatory integrity in response to low cardiac output and altered vascular capacitance in chronic HF (11, 12), or more simply serves a biomarker role reflecting disease progression remains a relevant question. In addition, the extent of BV expansion, the variability in the volume response, and the potential clinical impact of differing intravascular volume profiles on HF outcomes are yet undefined. Accordingly, we sought to quantify BV expansion, its variability, and relationship to clinical outcomes of morbidity and mortality in a cohort of patients with chronic HF. Our study hypothesis was that there would be significant heterogeneity in BV profiles at hospital discharge, but persistent BV expansion relative to normal BV would be frequent and supportive, not detrimental, overtime of better HF-related outcomes.

## METHODS

### Study Group

Nonconsecutive patients with known chronic HF admitted to the hospital for symptomatic clinically determined congestion, had undergone aggressive diuretic therapy, were considered decongested (euvolemic) by clinical criteria at the time of hospital discharge (resolved peripheral edema, reduced jugular venous distension, clear lungs on auscultation, less dyspnea with ambulation, and improved walking distance), and had undergone quantitative BV analysis (BVA) before discharge were followed prospectively. Patients were identified for the study from those admitted to hospital through the emergency department or from the outpatient of Mayo Heart Failure Clinic and met inclusion/exclusion criteria (see *Quantitation of Intravascular Volume*). Although BVA is a clinically available laboratory test, it may not be used in standard of care management in all patients with HF admitted by the primary cardiology care teams. All cohort patients were receiving standard oral HF medical therapy (β-blockers, angiotensin-converting enzyme inhibitors or angiotensin receptor blockers, mineralocorticoid receptor antagonists, and oral diuretics) at the time of discharge. Patient inclusion criteria were: *1*) age >18 yr, *2*) patients identified clinically with volume overload at the time of hospital admission with New York Heart Association functional Class III or IV status, *3*) ischemic or nonischemic cardiomyopathy HF etiology, and *4*) left ventricular ejection fraction (LVEF) measured within 6 mo before index hospitalization. Exclusion criteria: *1*) chronic kidney disease (CKD) requiring hemodialysis or undergoing ultrafiltration, *2*) known renal artery stenosis, nephrosis, or cirrhosis, *3*) females who were pregnant or of childbearing potential, and *4*) allergy to iodine refractory to medical management. Patients requiring intensive care unit therapy such as intravenous positive inotropes or vasodilators were not included in this study.

### Quantitation of Intravascular Volume

Quantitative blood volume analyses using the indicator-dilution method were undertaken in the Mayo Clinic Nuclear Medicine Laboratory using a standardized clinically available technique (Daxor BVA 100) to administer microcurie dose of iodinated I-131-labeled albumin intravenously. The specifics of the radiolabeled albumin indicator-dilution technique have been previously reported (8, 13, 14). Given that a “normal” BV in patients with chronic HF cannot be defined, volumes from nonHF normal individuals provide a frame of reference for the comparison of volume data. Normal volumes have been established based upon measurements in a broad population of healthy individuals of diverse body composition (including individuals with obesity) adjusted for height, weight, sex, and age (15, 16). For the purpose of this analysis, a reference normal BV was defined pre hoc for each individual patient as a measured volume greater than or equal to −8% to less than or equal to +8% of normal expected volume, mild-moderate expansion greater than +8% to less than +25%, and large BV expansion as greater than or equal to +25% above normal volume based upon previously reported data (8, 15, 16).

Red blood cell mass (RBCm) was calculated using mean whole body hematocrit (mWBHct) corrected from peripheral venous hematocrit and adjusted for trapped plasma to derive the measure of RBCm {[plasma volume (PV)/1−mWBHct] × mWBHct = RBCm}. The BV quantitative technique has been validated against double-labeled chromium-tagged red blood cells (RBCs) and I-125 albumin with comparisons within ±1% (17, 18). RBCm was evaluated to identify true anemia (defined as RBCm less than −10% of expected volume), normal RBCm (greater than or equal to −10% to less than or equal to +10% of normal volume), and polycythemia (defined as RBCm greater than +10% of normal volume). Measured intravascular volumes are reported as absolute values (in liters) and as a percent deviation [deficit (−) or excess (+)] from referenced normal volumes.

### Statistics

Data are presented as means ± standard deviation (SD) unless otherwise specified. To assess differences, volume variables were compared using Wilcoxon sign-rank test for paired analyses and percentage differences by χ^2^ analysis. Median values were compared using the Mann–Whitney *U* test. One-way ANOVA was used to test for significant differences between groups with *P* < 0.05 being considered statistically significant. Event-free survival was estimated using the Kaplan–Meier (K-M) log-rank analysis to test the differences in outcomes for the composite end point of HF-related mortality or first HF-related rehospitalization (postindex hospitalization) among the groups. Cox proportional hazards regression was used to assess the association of total BV and RBCm profiles with the time to posthospital composite outcome end point. Due to the limited number of events, univariate models were fit and variables that had a statistically significant association with the composite event were included in separate multivariable models with BV expansion and RBCm to assess the effect of total BV and RBCm profiles on outcomes after adjusting for relevant comorbidities. Results are presented as risk ratios (RRs) with 95% confidence intervals and *P* values.

Patient survival or HF-related mortality was confirmed using the Mayo Clinic, Rochester, electronic medical records system and social security death certification. First, postindex HF-related rehospitalization was identified by surveillance of electronic medical records and heart failure-related ICD-9-CM codes 425 and 428 and ICD-10-CM I50 codes. Median follow-up time was 8.2 mo with a minimum of 3 mo from the index hospital dismissal date. Study censor date for the subjects without a composite event was March 31, 2020. No deaths occurred during the index hospitalization, and no subjects were lost to follow-up. Renal function was expressed as the estimate glomerular filtration rate (eGFR), mL/min/1.73 m^2^, using the modification of diet in renal disease equation (19). Statistical analyses were performed using SAS, version 9, statistical software (SAS Institute, Cary, NC) and JMP 14.1 with *P* values <0.05 considered statistically significant. All participants provided written informed consent, and the study protocol was approved by the Mayo Foundation Institutional Research Review Board and Ethics Committee.

## RESULTS

### Blood Volume Profiles and Clinical Outcomes

Blood volume quantitation and clinical data were available on 110 patients who were prospectively followed for the composite end point over 1 year after the index hospitalization; 53 composite outcome events (33 deaths) occurred over the 1 year follow-up period. As described earlier, the cohort was stratified based upon the three BV profiles. The cut point of greater than or equal to +25% above the normal reference volume defined large expansion in BV (*n* = 56; 51% of cohort). A normal BV was identified in 30 patients (27% of cohort) and mild to moderate volume expansion in 24 patients (22% of cohort). No patients demonstrated a contraction in total BV. All patients were determined by the primary hospital service to be clinically decongested and for this study were blinded to discharge BVA findings. Table 1 shows the clinical and demographic features of the three subgroups at the time of hospital discharge. As shown, more males than females across the subgroups participated in the study, and patients with the greater BV expansion had lower fasting glucose level than patients with less blood volume expansion. The three subgroups were otherwise comparable with no differences in clinical characteristics, prevalence of comorbidities, duration of HF diagnosis, medications, laboratory values, or change in body weight in response to diuretic intervention during hospitalization. Table 1 also shows the absolute total BV, plasma volume (PV), RBCm, and percent deviation of these volumes from normal reference volumes. As expected, total BV was greater in the subgroup with volume expansion greater than or equal to +25%. RBCm and PV were also both significantly greater in this subgroup (70% demonstrated RBC polycythemia compared with only 15% in patients with total BV less than +25% expansion). RBCm was, therefore, a significant contributor to the increase in total BV along with moderate expansions in PV.

**Table 1.** Clinical and demographic characteristics stratified by TBV at hospital discharge

Variable	Normal TBV	Mild–Mod TBV Expansion	Large TBV Expansion, ≥+25%	*P* Value, Normal vs. ≥+25%	*P* Value, Normal vs. Mild–Mod
*n*	30	24	56		
Age, yr	67 ± 14	64 ± 14	70 ± 12	0.300	0.882
Sex, men/women	17/13	16/8	46/10	0.013	0.457
Body mass index, kg/m²	32 ± 7	34 ± 9	32 ± 7	1.00	0.362
Systolic blood pressure, mmHg	113 ± 14	113 ± 18	114 ± 16	0.774	1.00
LVEF, %	35 ± 19	37 ± 19	34 ± 16	0.797	0.702
Range	10–70	14–70	12–66		
RV systolic dysfunction, % ≥ moderate	40	42	60	0.080	0.883
Change in body wt during hospitalization, kg	−5.5 ± 5.0	−8.5 ± 7.7	−6.8 ± 4.4	0.211	0.090
Length of hospital stay, days	5.0 ± 1.6	5.7 ± 2.1	5.4 ± 1.7	0.292	0.170
Percentage (%) with					
Diabetes	53	62	39	0.215	0.511
Hypertension	73	71	63	0.352	0.872
Coronary artery disease	60	50	56	0.722	0.467
Atrial fibrillation	40	33	48	0.480	0.560
Sleep apnea	63	42	70	0.511	0.128
Hemoglobin, g/dL	11.7 ± 1.9	12.2 ± 2.1	11.8 ± 2.3	0.543	0.205
Creatinine, mg/dL	1.7 ± 0.7	1.9 ± 1.3	1.8 ± 0.7	0.530	0.473
eGFR, mL/min/1.73 m^2^	46 ± 23	45 ± 28	45 ± 21	0.839	0.886
BUN, mg/dL	38 ± 17	43 ± 29	42 ± 21	0.372	0.433
Sodium, mEq/L	140 ± 3.6	138 ± 4.7	139 ± 4.0	0.256	0.082
Potassium, mEq/L	4.1 ± 0.4	4.3 ± 0.9	4.1 ± 0.5	1.00	0.074
Plasma albumin, g/dL	3.6 ± 0.6	3.7 ± 0.4	3.6 ± 0.4	1.00	0.487
Plasma glucose, mg/dL	139 ± 52	137 ± 46	109 ± 21	0.001	0.883
NT-proBNP, pg/mL, Median (IQR)	5,001 (2,404, 10,475)	3,667 (1,422, 10,036)	6,555 (3,444, 11,616)	0.384	0.401
Blood volume, L	5.2 ± 0.8	6.6 ± 1.2	7.6 ± 1.0	<0.001	<0.001
Range	3.2–6.9	4.8–8.9	4.8–10.1		
%Excess (+)/deficit (−)	+1.2 ± 7.1	+17 ± 4	+47 ± 16	<0.001	<0.001
Range	−10 to +10	+11 to +25	+26 to +113		
RBC mass, L	1.7 ± 0.4	2.2 ± 0.6	2.6 ± 0.7	<0.001	<0.001
Range	0.8–2.6	1.4–4.3	1.4–5.1		
%Excess (+)/deficit (−)	−14 ± 16	+3.3 ± 18	+23 ± 29	<0.001	<0.001
Range	−44 to +24	−30 to +41	−32 to +107		
Plasma volume, L	3.5 ± 0.6	4.3 ± 0.9	5.1 ± 0.9	<0.001	<0.001
Range	2.4–5.2	3.1–6.3	3.1–7.4		
%Excess (+)/deficit (−)	+11 ± 10	+26 ± 12	+58 ± 26	<0.001	<0.001
Range	−9 to +34	+5 to +52	+4 to +125		
Percentage (%) with					
Normal RBC mass	40	54	18	0.027	0.310
Anemia	57	17	12	<0.001	<0.001
RBC polycythemia	3	29	70	<0.001	0.01
Percentage (%) with					
Normal PV	53	8	2	<0.001	<0.001
Mild–Mod PV expansion	43	38	7	<0.001	0.713
PV greater than or equal to +25%	4	54	91	<0.001	<0.001

Values are means ± SD; *n*, number of patients; percentages of categories; and ranges. BUN, blood urea nitrogen; eGFR, estimated glomerular filtration rate; IQR, interquartile ranges; LVEF, left ventricular ejection fraction; Mod, moderate; NT-proBNP, NH_2_-terminal pro-brain natriuretic peptide; PV, plasma volume; RBC, red blood cell; RV, right ventricular; TBV, total blood volume.

**Figure 1.**
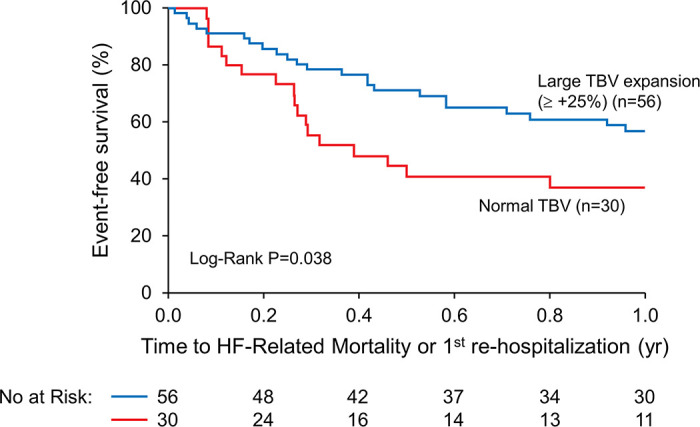
Total blood volume profiles and outcomes. Impact of blood volume expansion greater than or equal to +25% relative to normal blood volume on posthospital outcomes in patients with chronic heart failure—hospital discharge data. No. at Risk, number of patients at risk at each time point on the horizontal axis relating to the composite end-point outcome as indicated on the horizontal axis of the figures. Description of related statistical analyses, here Log-Rank analysis, are described in *Statistics*. HF, heart failure; TBV, total blood volume.

**Figure 2.**
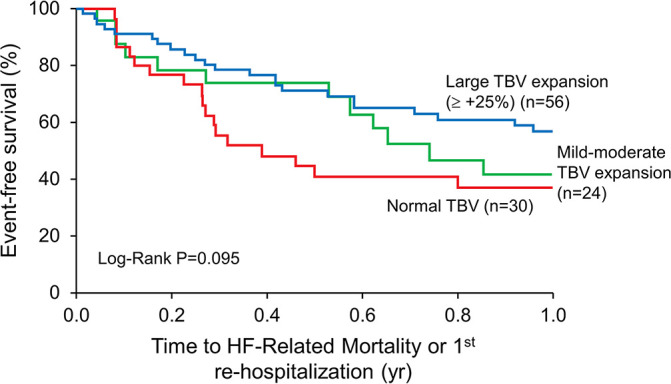
Impact of heterogeneity in blood volume profiles on clinical outcomes. Impact of blood volume profile heterogeneity on posthospital clinical outcomes in patients with chronic heart failure—hospital discharge data. HF, heart failure; TBV, total blood volume.

The results of clinical outcomes analyses for total BV greater than or equal to +25% compared with the patients with a normal BV are shown in Fig. 1 as K-M survival estimates for the composite end point (log-rank *P* = 0.038). This reveals distinctly different time courses to events with greater volume expansion (greater than or equal to +25%) demonstrating better outcomes relative to the risk associated with a normal BV at hospital discharge [RR 0.53 (0.28, 0.96), *P* = 0.044]. Of note, the curves show early divergence at ∼6 wk posthospitalization. Within the subgroup of BV expansion greater than or equal to +25%, the median degree of volume expansion was +42% [interquartile range (IQR) 34, 49%], reflecting an approximate 1.5–2-L expansion in intravascular volume from normal. To illustrate the interrelationship of all three subgroups, Fig. 2 shows the K-M survival curve data of the mild-moderate BV expansion subgroup added to Fig. 1 demonstrating an intermediate risk.

Table 1 also shows the frequency distribution of RBCm [normal RBCm, true (nondilutional) anemia, and RBC polycythemia] and PV (normal, mild-moderate expansion, and large expansion, greater than or equal to +25%) profiles in relation to the three BV subgroups. Patients with a normal overall BV at hospital discharge demonstrated a high prevalence of true anemia (57%) along with compensatory increases in PV to maintain an overall normal total BV. From another perspective, of patients with low hemoglobin (Hb; *n* = 40, as defined by World Health Organization (WHO) criteria of <12 g/dL for females and <13 g/dL for males), 60% (24/40) demonstrated true anemia, the remainder dilutional pseudo-anemia. In contrast, the BV subgroup with greater than or equal to +25% expansion revealed a high prevalence of RBC polycythemia (70%), as well as, PV expansion (greater than or equal to +25%) in 91%.

### Relation of RBC Mass to Outcomes

Given the earlier findings of increased RBCm contributing substantially to total BV expansion and recognizing that RBCm, as well as PV, constitute total BV, we assessed the association of RBCm and its variability to the composite outcome. The patient cohort was then stratified by normal RBCm, true anemia, and RBC polycythemia as defined earlier. Table 2 shows the clinical and demographic features of the overall patient cohort when defined by these RBCm profiles. RBC polycythemia was the most common profile (43%) of the overall cohort with true anemia 25% and normal RBCm 32%. As expected, peripheral venous hemoglobin (Hb) and hematocrit were lowest in the true anemia subgroup, whereas plasma glucose was highest. Otherwise, no significant differences were identified across these subgroups. Table 3 displays absolute and relative volume data, showing significantly higher total BV in the polycythemia subgroup and significantly lower BV in the subgroup with an RBCm deficit. Clinically identified blood loss as a basis for low RBCm was not identified in any of the patients. Measured PV was not significantly different among the three RBCm subgroups, although there was a statistically nonsignificant trend for a higher (+ percent expansion) PV in the polycythemia subgroup relative to normal PV (*P* = 0.08). All three subgroups demonstrated marked variability (low to high) in extents of volume expansion.

**Table 2.** Clinical and demographic characteristics at hospital discharge stratified by RBC mass profiles

Variable	Normal RBCm*	RBC Polycythemia**	True Anemia***	Intergroup Comparison *P* Value
*n*	35	47	28	
Age, yr	65 ± 13	71 ± 12	68 ± 14	0.140
Sex, men/women	23/8	30/13	20/8	0.851
Body mass index, kg/m²	32 ± 8	32 ± 7	33 ± 9	0.849
Systolic blood pressure, mmHg	113 ± 16	113 ± 15	117 ± 19	0.550
Duration of heart failure, mo	39 ± 17	47 ± 31	47 ± 29	0.379
Length of hospital stay, days	5.2 ± 1.7	5.4 ± 2.0	5.4 ± 1.8	0.880
LVEF, %	35 ± 19	33 ± 17	40 ± 16	0.253
Range	10–70	14–70	15–67	
RV systolic dysfunction, % ≥ Mod	53	60	32	0.102
Percentage (%) with				
Diabetes	52	40	54	0.250
Hypertension	77	58	75	0.088
Coronary artery disease	59	49	71	0.069
Atrial fibrillation	42	51	36	0.218
Sleep apnea	52	67	68	0.212
Hemoglobin, g/dL	12.0 ± 1.7	13.0 ± 1.8	9.7 ± 1.3	0.001
Venous hematocrit, %	37.2 ± 4.8	41.6 ± 5.8	30.3 ± 4.3	<0.001
Serum creatinine, mg/dL	1.7 ± 0.7	1.6 ± 0.6	2.0 ± 1.2	0.134
eGFR, mL/min/1.73 m^2^	49 ± 21	49 ± 25	43 ± 23	0.510
BUN, mg/dL	39 ± 17	41 ± 21	46 ± 28	0.455
Plasma albumin, g/dL	3.7 ± 0.5	3.7 ± 0.4	3.4 ± 0.5	0.023
Sodium, mEq/L	139 ± 3.7	139 ± 3.9	139 ± 4.3	1.000
Potassium, mEq/L	4.1 ± 0.4	4.2 ± 0.4	4.2 ± 0.5	0.552
Plasma glucose, mg/dL	126 ± 35	110 ± 31	140 ± 51	0.001
NT-proBNP, pg/mL	4,387	5,487	5,946	0.552
Median (IQR)	(2,330, 13,472)	(2,620, 9,708)	(3,799, 10,549)	

Values are means ± SD; *n*, number of patients; percentages of categories; medians; and interquartile ranges (IQR). *Normal range red blood cell (RBC) mass greater than or equal to −10% to less than or equal to +10% of normal volume; **polycythemia: RBC mass greater than +10% of normal volume; ***true anemia: RBC mass less than −10% of normal volume. BUN, blood urea nitrogen; eGFR, estimated glomerular filtration rate; LVEF, left ventricular ejection fraction; Mod, moderate; NT-proBNP, NH_2_-terminal pro-brain natriuretic peptide; RBCm, red blood cell mass; RV, right ventricular.

**Table 3.** Total blood volume and PV at hospital discharge stratified by RBC mass profile

Variable	Normal RBCm*	RBC Polycythemia**	True Anemia***	Intergroup Comparison *P* Value
*n*	35	47	28	
Total blood volume, L	6.4 ± 1.3	7.4 ± 1.3	6.1 ± 1.6	<0.001
Range	4.1–8.4	4.8–10.1	3.2–9.1	
Total blood volume, %excess (+)/deficit (−)	+20 ± 17	+41 ± 20	+11 ± 18	<0.001
Range	−9 to +62	+7 to +113	−12 to +52	
Plasma volume, L	4.2 ± 1.0	4.6 ± 0.9	4.5 ± 1.3	0.258
Range	2.6–6.1	2.9–7.0	2.3–7.4	
Plasma volume, %excess (+)/deficit (−)	+34 ± 27	+45 ± 28	+33 ± 30	0.115
Range	−9 to +99	−3 to +125	−3 to +101	
Red blood cell mass, L	2.1 ± 0.3	2.8 ± 0.7	1.6 ± 0.4	<0.001
Range	1.2–2.7	1.6–5.1	0.8–2.7	
Red blood cell mass, %excess (+)/deficit (−)	+1.4 ± 6.4	+34 ± 22	−24 ± 9	<0.001
Range	−10 to +8.4	+11 to +107	−44 to −11	
Percentage with				
Normal TBV	34	2	61	0.021
Mild–mod TBV expansion	37	15	14	0.043
TBV ≥+25%	29	83	25	0.034
Percentage with				
Normal PV	23	9	25	0.087
Mild–mod PV expansion	23	15	36	0.100
PV ≥+25%	54	76	39	0.040

Values are means ± SD; *n*, number of patients; percentages of categories; and ranges. *Normal range red blood cell (RBC) mass (RBCm): greater than or equal to −10% to less than or equal to +10% of normal expected volume; **RBC polycythemia: RBCm greater than +10% of normal volume; ***true anemia: RBCm less than −10% of normal volume. Mod, moderate; PV, plasma volume; TBV, total blood volume.

**Figure 3.**
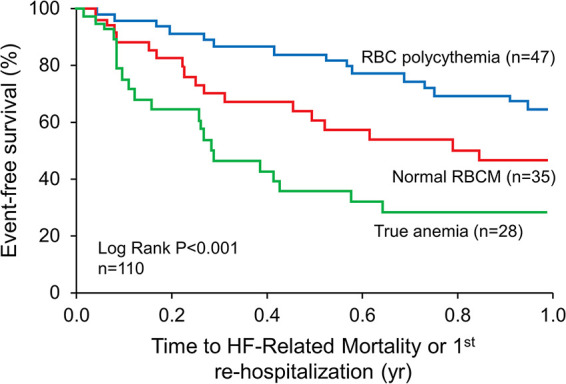
Red blood cell mass and clinical outcomes. Impact of RBC mass profiles at the time of hospital discharge on posthospital clinical outcomes in patients with chronic heart failure. HF, heart failure; RBC, red blood cell; RBCm, red blood cell mass.

K-M curve survival analysis (Fig. 3) also demonstrated distinctly different time courses for the composite outcome events over the 1-year posthospital follow-up period based upon the three RBCm profiles (log-rank: *P* < 0.001). Polycythemia demonstrated the best relative outcome [RR 0.39 (0.21–0.71), *P* = 0.002] and as indicated earlier was highly prevalent (70%) in patients with BV expansion greater than or equal to +25%. The patients with normal RBCm demonstrated an intermediate risk profile and those with true anemia had the poorest event-free survival. It is of value to note that typical clinical HF risk factors such as hypertension, diabetes, coronary artery disease, and elevated NH_2_-terminal pro-brain natriuretic peptide (NT-proBNP) level were not different across the three subgroups.

As shown in Table 1, peripheral venous Hb concentrations did not differ across the three BV subgroups despite significant differences in RBCm. In addition, in contrast to RBCm profiles, Hb concentration at the time of hospital dismissal stratified above and below the cohort median Hb value of 12 g/dL did not discriminate risk for the composite end point (log-rank *P* = 0.07, data not shown). Furthermore, in regression analysis, venous Hb did not reveal a clinically meaningful correlation with measured BV (*r* = 0.100, *P* = 0.352) or as percent deviation from normal BV (*r* = 0.050, *P* = 0.733).

### Univariate and Multivariable Analyses

Cox proportional hazards regression analysis was used to evaluate the association among hospital discharge BV profiles and HF-related mortality or first HF rehospitalization. All variables included in Table 1 were evaluated by univariate models, which demonstrated the only significant predictors in addition to the quantitated volume variables to be eGFR (*P* = 0.006) and hematocrit (*P* = 0.003).

In a model comprising BV expansion greater than or equal to +25% and mild-moderate BV expansion, the analysis demonstrated that BV expansion greater than or equal to +25% was significantly different from normal BV (*P* = 0.044). When the univariate predictor eGFR was entered into the base multivariable model of BV greater than or equal to +25% plus mild-moderate BV expansion (Total BV Model, Table 4), higher eGFR and BV expansion greater than or equal to +25% remained independent predictors of better event-free survival. Because of concerns for colinearity with RBCm, hematocrit was not included in the multivariable modeling.

**Table 4.** Univariate and multivariable Cox proportional hazard model analyses for relative risk of posthospital HF-related mortality or first HF-related rehospitalization

Variable	Univariate Model Risk Ratio (95% CI)	*P* Value	Multivariable Model Risk Ratio (95% CI)	*P* Value
Total BV model				
BV expansion ≥+25%	0.53 (0.28–0.96)*	0.044	0.52 (0.28–0.96)	0.037
Mild–Mod BV expansion	0.96 (0.50–1.86)*	0.908	0.83 (0.39–1.77)	0.632
eGFR	0.14 (0.03–0.58)	0.006	0.15 (0.03–0.69)	0.022
RBC mass model				
RBC polycythemia	0.39 (0.21–0.71)**	0.002	0.44 (0.24–0.77)	0.003
True anemia	2.7 (1.54–4.64)**	<0.001	2.25 (1.30–3.82)	0.005
eGFR	0.14 (0.03–0.58)	0.006	0.16 (0.04–0.69)	0.014

Values are ratios and confidence intervals (CI). *Relative risk (RR) is relative to normal blood volume (BV); **RR is relative to normal red blood cell (RBC) mass. eGFR, estimated glomerular filtration rate; HF, heart failure; Mod, moderate.

In univariate regression modeling for RBCm, both RBC polycythemia (RBCm excess) and true anemia (RBCm deficit) were significant predictors but with opposing directions of risk (RBC Mass Model, Table 4). A model of these variables demonstrated that both were different from normal RBCm (*P* = 0.001). With RBC polycythemia and true anemia as the base model and then entering separately the univariate predictor eGFR, RBC polycythemia (better outcome) and true anemia (worse outcome) remained strong predictors along with higher eGFR (better outcome) of the composite end point (Table 4).

## DISCUSSION

The principal findings of this study are the following: first, possibly contrary to clinical expectations, persistent BV expansion rather than a normal intravascular volume was an independent predictor of higher event-free survival and lower rehospitalization rate in this cohort of patients with stable advanced chronic HF. Although the prospective observational design of this study does not permit a cause and effect relationship to be established, BV expansion would be expected to be a mechanism to help maintain an effective circulatory system in the context of HF with impaired cardiac output and relative vascular underfilling with altered venous capacitance, particularly vascular remodeling of the splanchnic venous reservoir. This observation also has support in findings of earlier reported human studies (1–7, 20, 21). The current results also indicate that significant BV expansion appears to be a highly prevalent and persistent volume profile even after intensive in-hospital intravenous diuretic therapy. However, it is important to recognize that there is also marked variability in intravascular volume profiles, and BV expansion does not appear to be a uniform physiological response in patients with chronic HF. Furthermore, as shown here for the first time, the identified variability in BV profiles is significantly associated with clinically significant differences in HF-related morbidity and mortality outcomes. The basis for the more favorable outcomes with volume expansion would physiologically relate to the priority of maintaining an effective circulating BV such that an appropriate filling of the vascular system can support an appropriate Frank–Starling relationship of preload, cardiac output, and perfusion pressure. In this context, advocating guideline-recommended treatment to “euvolemia” might be interpreted as identifying and managing patients to an optimal BV (“intravascular euvolemia”), which based upon the current findings requires intravascular volume expansion. This is hypothesis generating and requires testing by a randomized controlled trial. Also, consistent with maintaining circulatory integrity and still effecting clinical decongestion (improvement in signs and symptoms of HF) are the reported observations that net fluid loss resulting from diuretic therapy is derived almost entirely from the interstitial compartment with the intravascular compartment demonstrating only minor changes in volume (3, 4, 6, 22).

Second, a normal BV (absence of any expansion in BV) was present in a minority of patients being observed in only 27% of the cohort at hospital discharge and was associated with significantly worse event-free survival relative to BV expansion. Whether this reflects a failure in renal and/or neuroendocrine responses, a less effective splanchnic venous volume reservoir response (23, 24) to regulate intravascular volume, or limitations in other compensatory responses perhaps genotype based cannot be established from this analysis but is an area that merits further investigation.

A third observation relates to the high prevalence and clinical significance of an increased RBCm (RBC polycythemia) particularly in relation to peripheral Hb concentrations. RBC polycythemia was identified in 43% of this cohort with additional reports of high prevalence in patients hospitalized for decompensated HF (1, 2, 25–27). RBC polycythemia was also associated with better clinical outcomes and likely serves a compensatory physiological role to enhance oxygen-carrying capacity in patients with chronic HF. It is also appropriate to note that RBC polycythemia was accompanied by a compensatory increase in PV, which would have importance in mitigating any potential for viscosity or thrombosis risk. Although measured absolute PV was not different on average among the three RBCm profiles (Table 1), a large expansion in PV (greater than or equal to +25% above normal) was identified in the majority (76%) of the subgroup with RBC polycythemia (Table 3). This emphasizes the significance of not only total BV expansion but also the components of volume expansion, which includes the contribution of RBCm. This is underscored by the observation that in the subgroup with total BV greater than or equal to +25%, 70% demonstrated RBC polycythemia, whereas only 18% demonstrated a normal RBCm and 12% true anemia. Thus, RBC polycythemia may be an attribute favoring better outcomes in response to chronic tissue hypoxia as can occur in HF-related CKD (28–30) or hypoxia secondary to sleep apnea. Our data are hypothesis-generating from this perspective and more investigation is needed to better understand the mechanism(s) driving the development of RBC polycythemia, its potential beneficial role, and possible risks in relation to the overall heterogeneity in BV profiles and clinical outcomes.

Fourth, contrasting RBC polycythemia, true anemia (RBCm deficit) was identified in 23% of the overall cohort and was associated with less volume expansion and an increase in risk relative to normal RBCm. It is, however, relevant to note that of the patients with normal and mild-moderate BV expansion (i.e., less than +25%, *n* = 54), only 21 (39%) demonstrated a deficit in RBCm, suggesting that true anemia itself was not the only driver of poorer outcomes in this BV subgroup. Also, although 61% of patients with true anemia had a normal overall total BV due to compensatory increases in PV, the degree of PV expansion itself was not sufficient to support better outcomes. The development of RBCm deficit is likely multifactorial, possibly from treatment effects, chronic disease effects to include CKD, occult blood loss, and/or limitations in compensatory mechanisms, but given its significance as a risk factor, inadequate or refractory elements of RBC production and sources of blood loss should be thoroughly evaluated as standard of HF care to include assessment for iron deficiency with or without concurrent anemia. In addition, evaluating RBCm to accurately detect true anemia as opposed to using peripheral Hb concentrations alone to identify anemia is also supported in the findings of this and previous analyses (27). Hemoglobin concentration is subject to the dilutional effects of PV expansion, thereby potentially providing an inaccurate picture of RBCm status and the presence or absence of true anemia.

### Limitations

First, this is an observational, single-center study of prospectively collected data from a tertiary referral medical center with potential selection bias and cannot establish cause and effect. Second, the patient sample demonstrated a preponderance of male subjects, and although this was the result of random chance of clinical presentation, it does reflect a bias for sex inclusion in the analysis. However, there were no differences in clinical outcomes demonstrated by within-group analysis based upon sex (all *P* ≥ 0.415). Therefore, the findings of this study reflect data from typical patients with chronic HF in a compensated state at the time of discharge with posthospital follow-up and, therefore, would be expected to have characteristics and features comparable with patients in other communities. Third, the generalization of the findings to all classes of patients with HF and suspected volume overload should not be done. This analysis focused on patients with more advanced HF, and it would not be expected that all stages and severity of HF would respond in the same way with mechanisms of volume regulation or compensation. This will require additional study in appropriately defined cohorts of patient with HF with a working hypothesis that earlier identification of the variability in intravascular volume status may have benefit in altering the natural history of HF progression. Fourth, the lack of serial BV measurements over the 1-year time course of the study limits the ability to account for possible changes in BV status (crossover) that may occur. However, data from our group in patients with less severe chronic HF demonstrated stable BV profiles with no volume profile crossover after 1 year (9). Ahlgrim et al. (31) have also reported stable vascular volumes over 6 mo in patients with compensated chronic HF. Fifth, not addressed in this analysis is the mechanism(s) behind the observation of some patients with HF maintaining or developing BV expansion and RBC polycythemia, whereas others do not. This is a key issue in further understanding the pathophysiology of chronic HF. To this point, right heart hemodynamics (filling pressures) have been shown to not be consistent indicators of volume status (32, 33) and, therefore, central pressures cannot be assumed to be the driver of intravascular volumes. Thus, whether the heterogeneity in BV profiles reflects pathophysiological differences in volume regulation, unintended responses to therapy, dysregulation in neuroendocrine responses, or functional limits (exhaustion) of compensatory mechanisms is yet to be determined and warrants further analysis for potential benefits to improve patient outcomes.

### Conclusions

The findings of this study demonstrate that intravascular volume profiles in patients with chronic HF vary substantially from one patient with HF to another even when in a similar state of clinical compensation. Furthermore and importantly, a volume profile of BV expansion (in contrast to a normal BV) is associated with higher event-free survival. In addition, RBC polycythemia contributes significantly to BV expansion, is common in advanced HF, and is independently associated with improved clinical outcomes. However, although BV expansion appears to be a common physiological response, it is not a uniform response with the marked heterogeneity in BV profiles clinically impacting significant differences in outcomes. These observations support BV expansion with RBC polycythemia as an underrecognized compensatory mechanism in chronic HF and highlight the biological complexity of intravascular volume regulation and the importance of identifying volume profiles not only to help stratify patient risk but also potentially to inform patient management. Also, such information can provide the basis for prospective clinical trials of volume-guided therapy versus standard of care volume management, which may help guide more individualized volume management strategies in patients with HF.

## GRANTS

This work was supported in part by the Mayo Clinic Department of Cardiovascular Medicine and unrestricted research grant from the Daxor Corporation, New York, NY, and Feldschuh Foundation for Clinical Research, New York, NY.

## DISCLOSURES

No conflicts of interest, financial or otherwise, are declared by the authors.

## AUTHOR CONTRIBUTIONS

W.L.M. conceived and designed research; W.L.M. and B.P.M. performed experiments; W.L.M. and D.E.G. analyzed data; W.L.M., J.E.S., and B.P.M. interpreted results of experiments; W.L.M. prepared figures; W.L.M. drafted manuscript; W.L.M. and J.E.S. edited and revised manuscript; W.L.M., J.E.S., D.E.G., and B.P.M. approved final version of manuscript.
